# Supplementary material for: Minimal barriers to invasion during human colorectal tumor growth
Source: Nat Commun. 2020 Mar 9;11:1280. doi: 10.1038/s41467-020-14908-7 (PMC7062901; doi:10.1038/s41467-020-14908-7)
Supplement: Supplementary file 1 — Supplementary Information [file 41467_2020_14908_MOESM1_ESM.pdf]

Supplementary Information to Manuscript

Minimal Barriers to Invasion During Human Colorectal Tumor Growth

Ryser et al.

Supplementary Table 1

ID	Type	Size (cm)	Stage	Slides	Public/ private mutations	SNV calls, n	Mixed spots, n	False positive SNV, n	False negative SNV, n	Mixed spots, ID	False positive SNV, ID	False negative SNV, ID
C	CRC	6.4	S3	2	27/20	912	-	-	-	-	-	-
D	CRC	2.0	S1	2	23/27	1881	1	2	2	A4_3	A4_38: TP53, RC3H1	A3_37: CD109, CCKBR
E	CRC	6.1	S1	2	54/36	2970	-	1	-	-	A3_13: HOXD1	-
F	CRC	1.8	S1	2	29/16	1643	1	2	-	A6_25	A5_12: IGSF10; A5_28: GRIK5	-
H	CRC	4.0	S4	2	12/10	1170	1	-	-	A11_17	-	-
J	CRC	5.0	S3	2	17/0	756	-	-	-	-	-	-
K	Adx	6.0	-	2	11/25	1189	-	-	-	-	-	-
M	CRC	3.0	S2	2	7/6	377	-	-	-	-	-	-
R	CRC	3.5	S1	1	36/6	870	-	4	-	-	A7_7: SLC1A3, CENPE, ARL8B, SMARCE1	-
T	CRC	5.7	S3	2	22/5	910	1	2	-	A7_1	A10_7: SLC8A1, DOPEY1	-
U	CRC	3.9	S2	2	25/3	667	-	-	-	-	-	-
W	CRC	3.4	S1	2	59/9	2040	-	1	-	-	A6_24A: ZNF23	-
**Total**				**23**	**322/163**	**15,385**	**4**	**12**	**2**			

Supplementary Table 1: Homoplasy avoidance for phylogenetic tree construction. After pre-processing (see Methods), tumor-specific modifications were made to avoid homoplasy: removal of non-clonal spots (genotype mixtures); removal of false positive mutation calls of low allele frequency; and addition of false negative calls of low allele frequency. SNV: single nucleotide variant.

Supplementary Figure 1. Variant allele frequency (VAF) distributions among microdissected spots. (a) Spots negative for mutations had a VAF less than 0.05 (average of 0.002). **(b)** Average VAF for private mutations (present in a strict subset of spots) was 0.343. **(c)** Average VAF for public mutations (present in all spots) was 0.374. Public mutations may be present on more than one chromosome (i.e. homozygous) and there was no significant difference (p>0.05, t-test) between the public and private mutations when the public mutations with frequencies greater than 0.65 were excluded. The similar public and private mutation frequencies in the microdissected spots indicate near clonal subpopulations. SNV: single nucleotide variants.

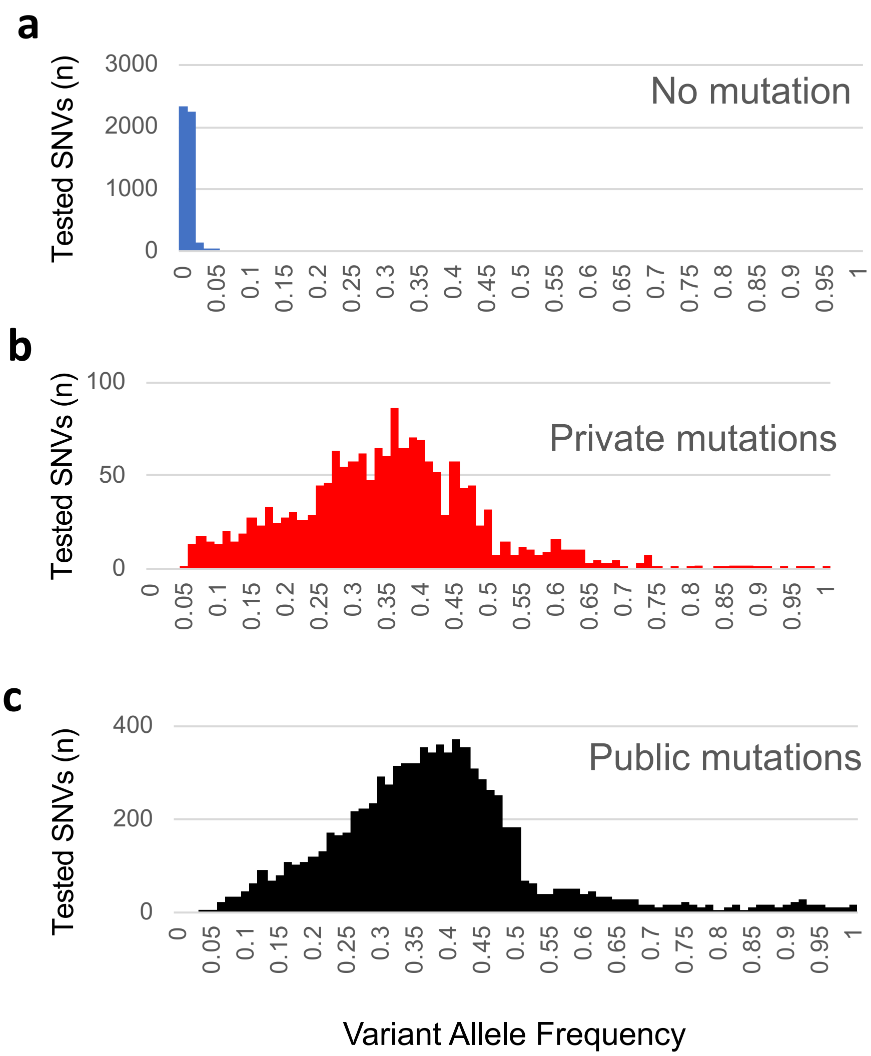

Supplementary Figure 2: Phylogeographies of 6 tumors. (a) Spot genotypes were identified and localized on the sections, and sections were annotated for superficial and invasive regions. Invasion was defined as migration past the muscularis mucosae, with the likely original location noted by the dotted line. The sole adenoma (tumor K) had focal stalk invasion, which may represent the start of deeper invasion. **(b)** Contiguous clone maps were derived from the spot topographies, revealing vertically arranged subclones that share superficial and invasive phenotypes. **(c)** Maximum parsimony algorithms were used to reconstruct phylogenetic trees for the subclones. **(d)** The ancestry of invasive subclones was reconstructed under the assumption that cells migrate from superficial to invasive regions, but not vice-versa.

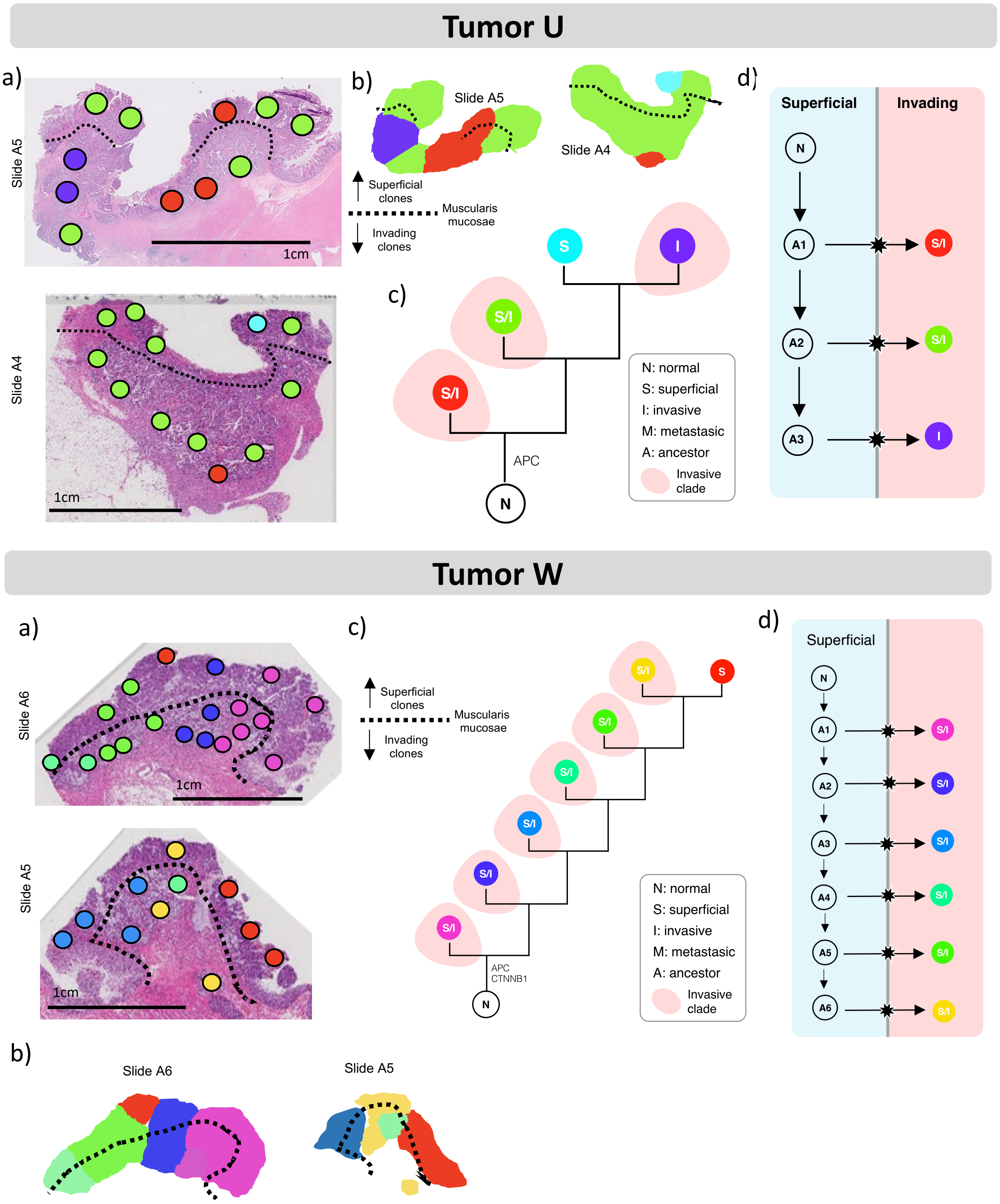

Tumor F

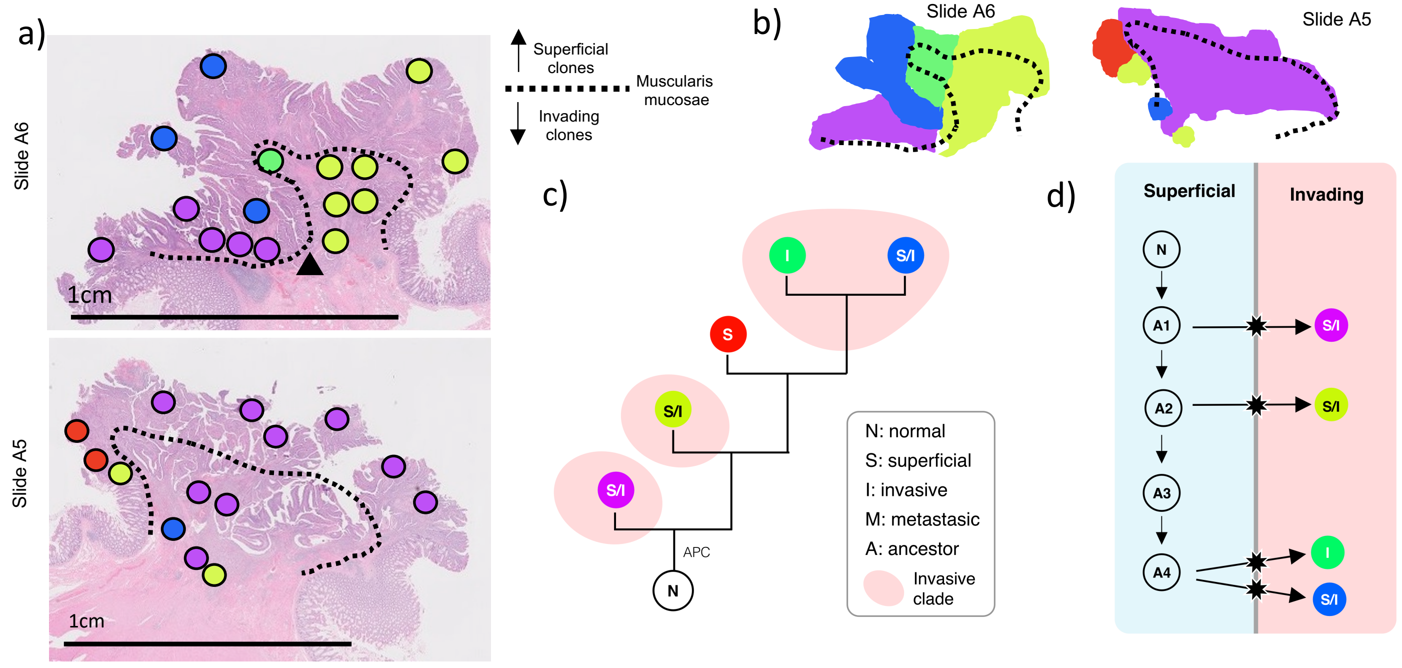

Tumor T

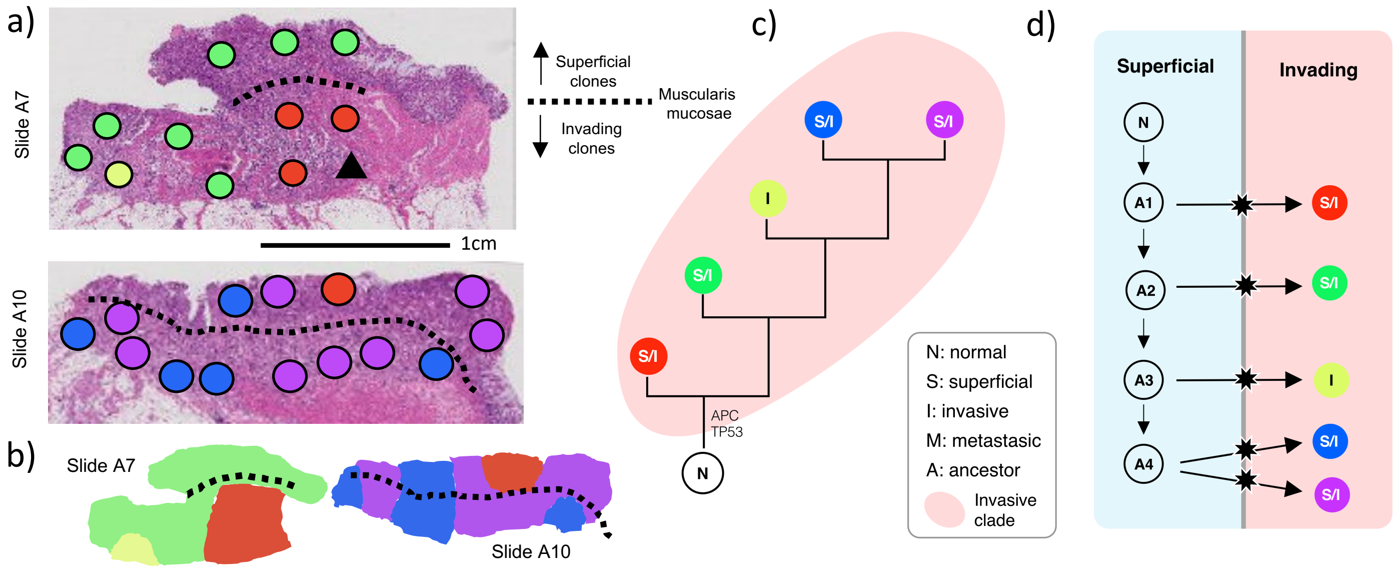

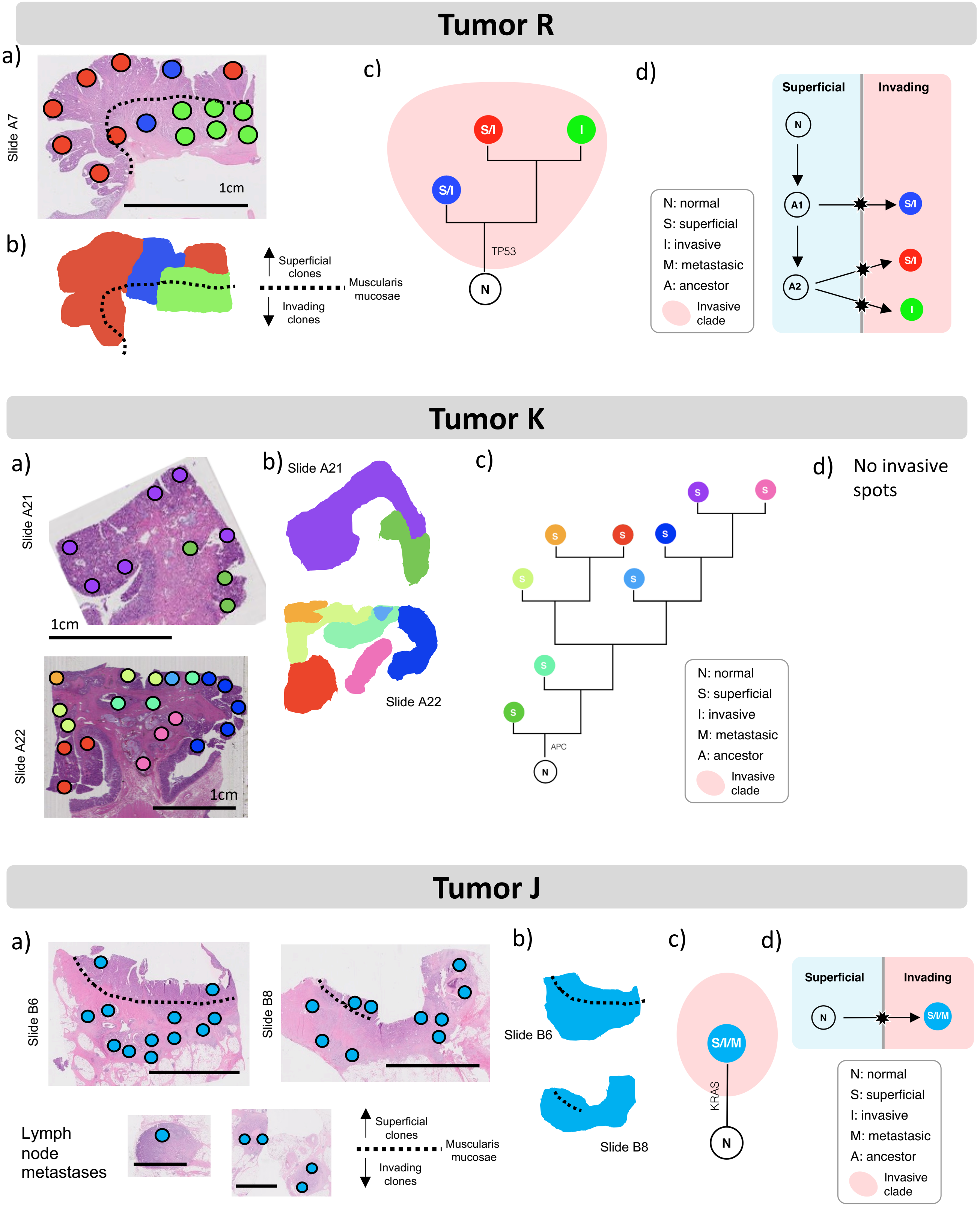

Tumor R
a)
Slide A7
1cm
b)
Superficial clones
Muscularis mucosae
Invading clones
c)
S/I
I
S/I
TP53
N
d)
N: normal
S: superficial
I: invasive
M: metastasic
A: ancestor
Invasive clade
Superficial
Invading
N
A1
S/I
A2
S/I
I
Tumor K
a)
Slide A21
1cm
Slide A22
1cm
b) Slide A21
Slide A22
c)
S
S
S
S
S
S
S
S
S
APC
N
N: normal
S: superficial
I: invasive
M: metastasic
A: ancestor
Invasive clade
d) No invasive spots
Tumor J
a)
Slide B6
Slide B8
Lymph node metastases
Superficial clones
Muscularis mucosae
Invading clones
b)
Slide B6
Slide B8
c)
S/I/M
KRAS
N
d)
Superficial
Invading
N
S/I/M
N: normal
S: superficial
I: invasive
M: metastasic
A: ancestor
Invasive clade

Supplementary Figure 3: t-SNE plots. Nonlinear embedding (t-SNE) of the sequencing data illustrates clustering properties of spots by genotype (colors) and phenotype (shape). met: metastasis

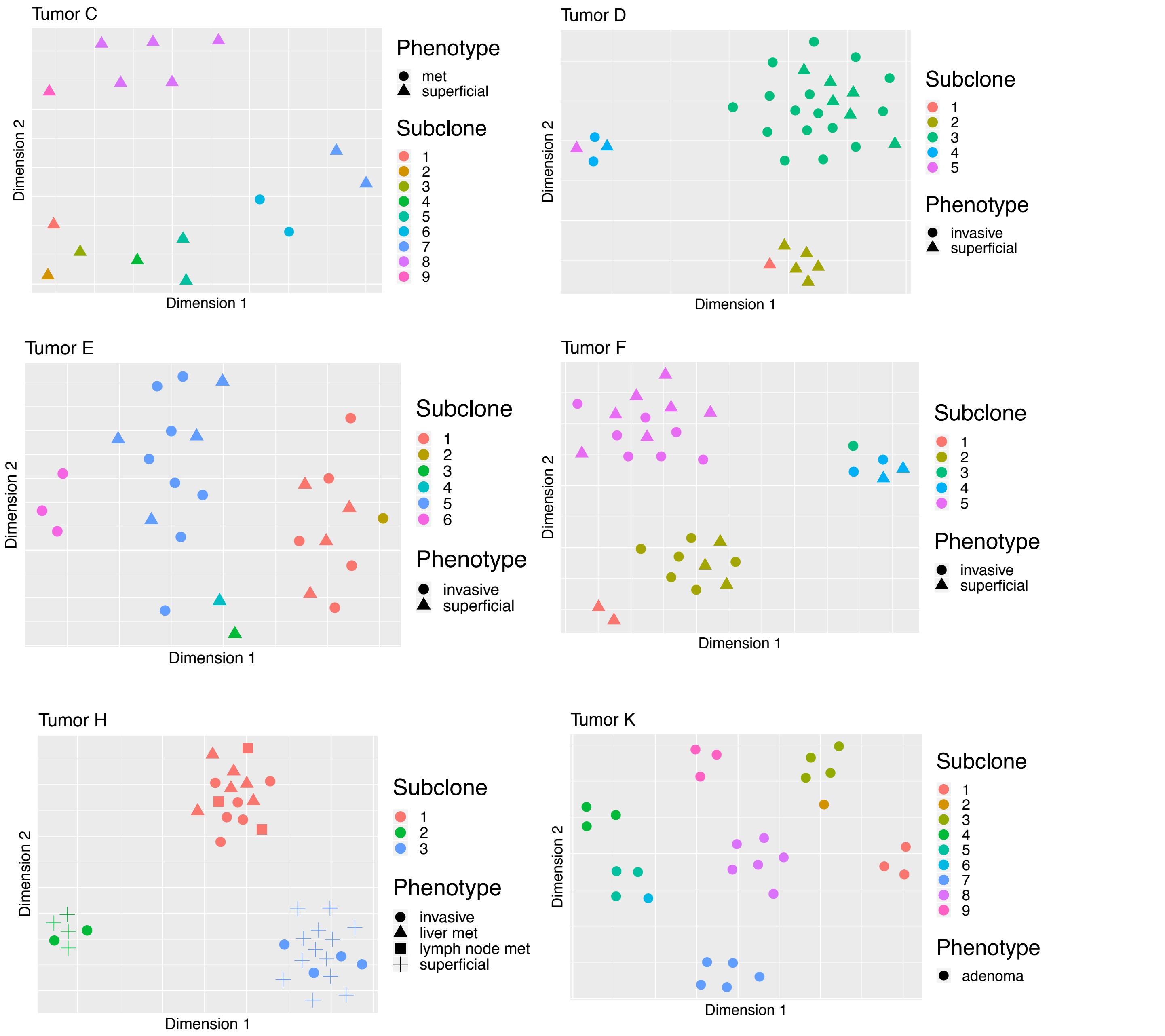

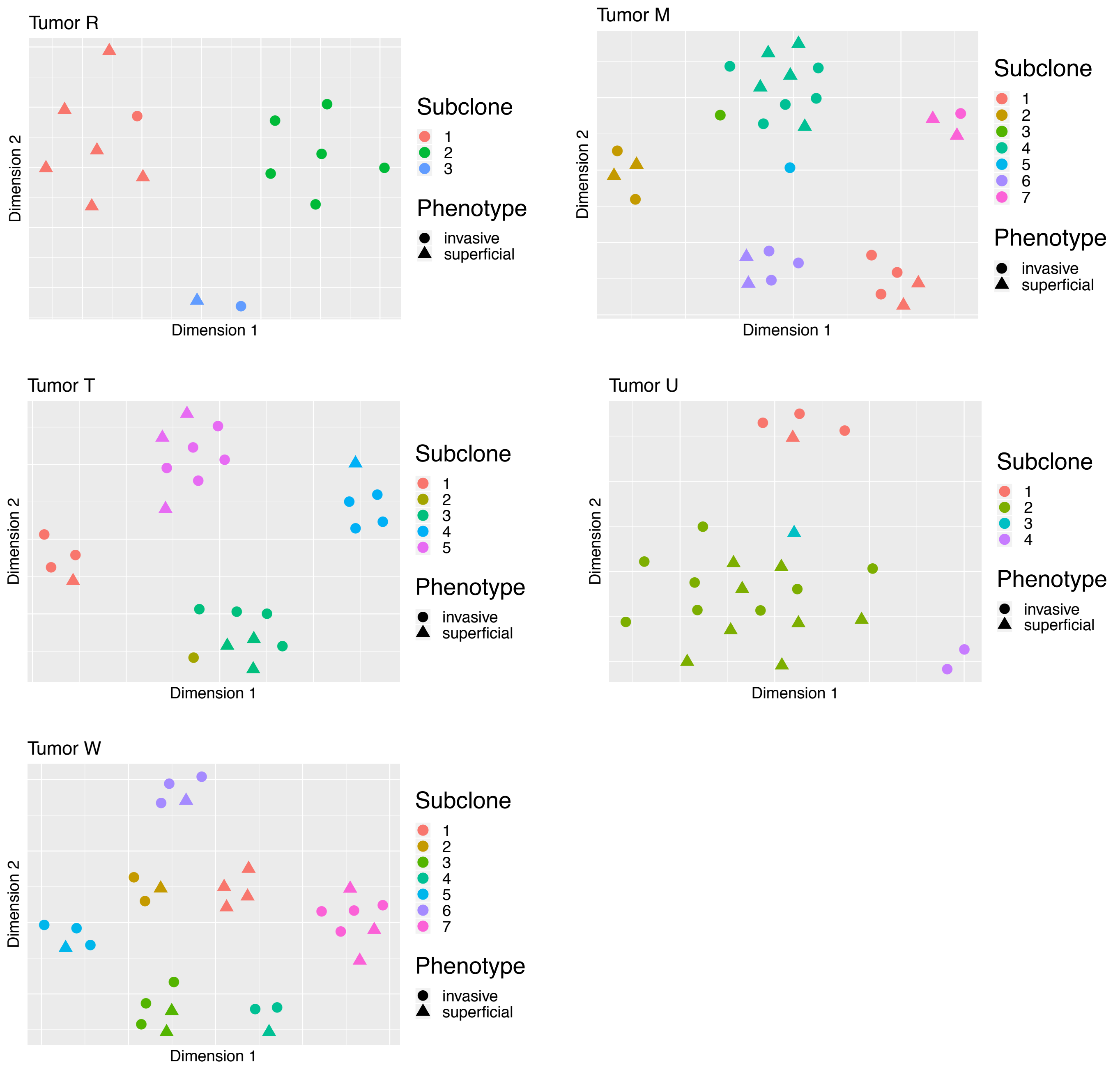
Tumor R
Dimension 2
Dimension 1
Subclone
1
2
3
Phenotype
invasive
superficial
Tumor M
Dimension 2
Dimension 1
Subclone
1
2
3
4
5
6
7
Phenotype
invasive
superficial
Tumor T
Dimension 2
Dimension 1
Subclone
1
2
3
4
5
Phenotype
invasive
superficial
Tumor U
Dimension 2
Dimension 1
Subclone
1
2
3
4
Phenotype
invasive
superficial
Tumor W
Dimension 2
Dimension 1
Subclone
1
2
3
4
5
6
7
Phenotype
invasive
superficial

Supplementary Figure 4. Annotated clone maps and phylogeographies for all 12 tumors. (a) Annotated slides with spot labels. **(b)** Mutation calls after tree reconstruction pre-processing (see Methods). Rows: mutations; columns: spots. Purple: Mutation present; blue: mutation absent. Subclone names (C1, C2, …) indicated at bottom of panel. **(c)** Rooted maximum parsimony tree with root on left; x-axis represents phylogenetic distance between subclones (different colors). **(d)** Phylogenetic tree (not to scale) showing subclone name as identified in panel b. Shaded red regions indicate invasive events. The ancestral tree combines information from a previous study[1] where the dotted and dashed branches indicate the locations of the private mutations found on opposite tumor sides. The switching of lineages indicates early cell mixing during growth[1].

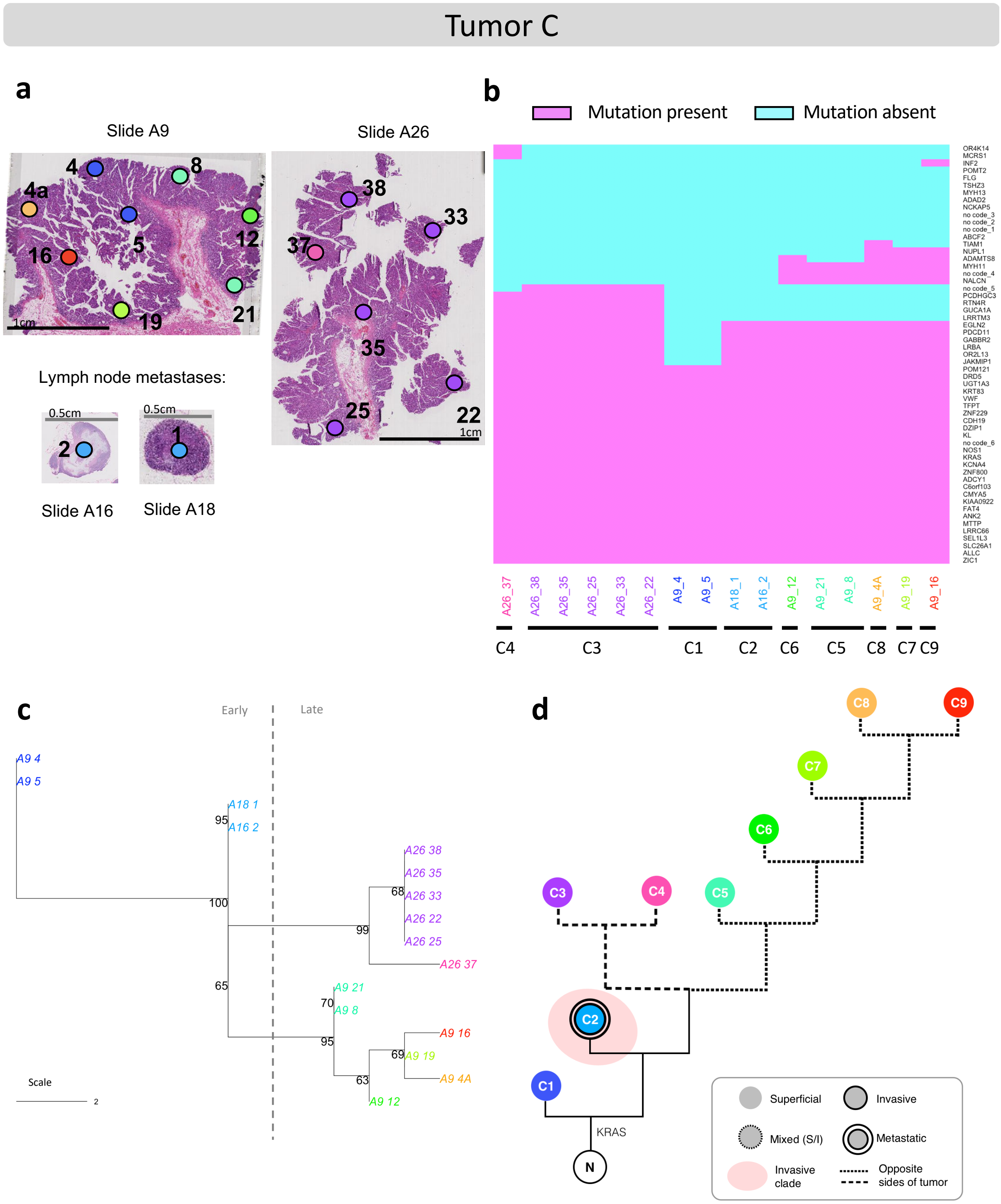

Tumor D

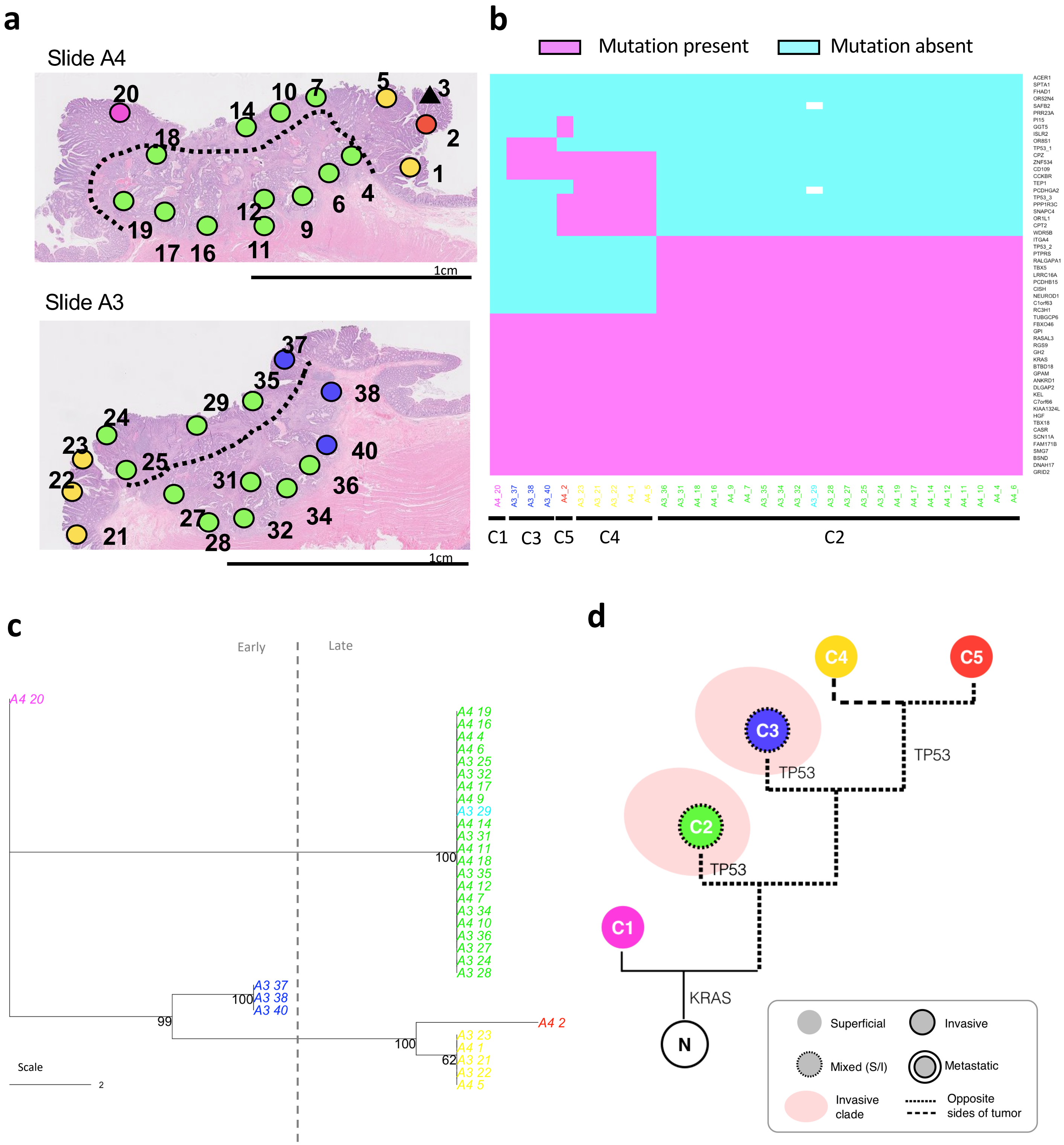
a
Slide A4
20 18 14 10 7 5 3 2 1 4 6 9 12 11 19 17 16
1cm
Slide A3
37 35 38 29 24 23 25 22 40 31 36 27 28 32 34 21
1cm
b
Mutation present
Mutation absent
ACER1
SPTA1
FHAD1
OR52N4
SAFB2
PRR23A
PI15
GGT5
ISLR2
OR8S1
TP53_1
CPZ
ZNF534
CD109
CCKBR
TEP1
PCDHGA2
TP53_3
PPP1R3C
SNAPC4
OR1L1
CPT2
WDR5B
ITGA4
TP53_2
PTPRS
RALGAPA1
TBX5
LRRC16A
PCDHB15
CISH
NEUROD1
C1orf63
RC3H1
TUBGCP6
FBXO46
GPI
RASAL3
RGS9
GH2
KRAS
BTBD18
GPAM
ANKRD1
DLGAP2
KEL
C7orf66
KIAA1324L
HGF
TBX18
CASR
SCN11A
FAM171B
SMG7
BSND
DNAH17
GRID2
A4_20 A3_37 A3_38 A3_40 A4_2 A3_23 A3_21 A3_22 A4_1 A4_5 A3_36 A3_31 A4_18 A4_16 A4_9 A4_7 A3_35 A3_34 A3_32 A3_29 A3_28 A3_27 A3_25 A3_24 A4_19 A4_17 A4_14 A4_12 A4_11 A4_10 A4_4 A4_6
C1 C3 C5 C4 C2
c
Early
Late
A4 20
A4 19
A4 16
A4 4
A4 6
A3 25
A3 32
A4 17
A4 9
A3 29
A4 14
A3 31
A4 11
A4 18
A3 35
A4 12
A4 7
A3 34
A4 10
A3 36
A3 27
A3 24
A3 28
100
A3 37
A3 38
A3 40
100
99
A4 2
A3 23
A4 1
A3 21
A3 22
A4 5
100
62
Scale
2
d
C4
C5
C3
TP53
TP53
C2
TP53
C1
KRAS
N
Superficial
Invasive
Mixed (S/I)
Metastatic
Invasive clade
Opposite sides of tumor

Tumor E

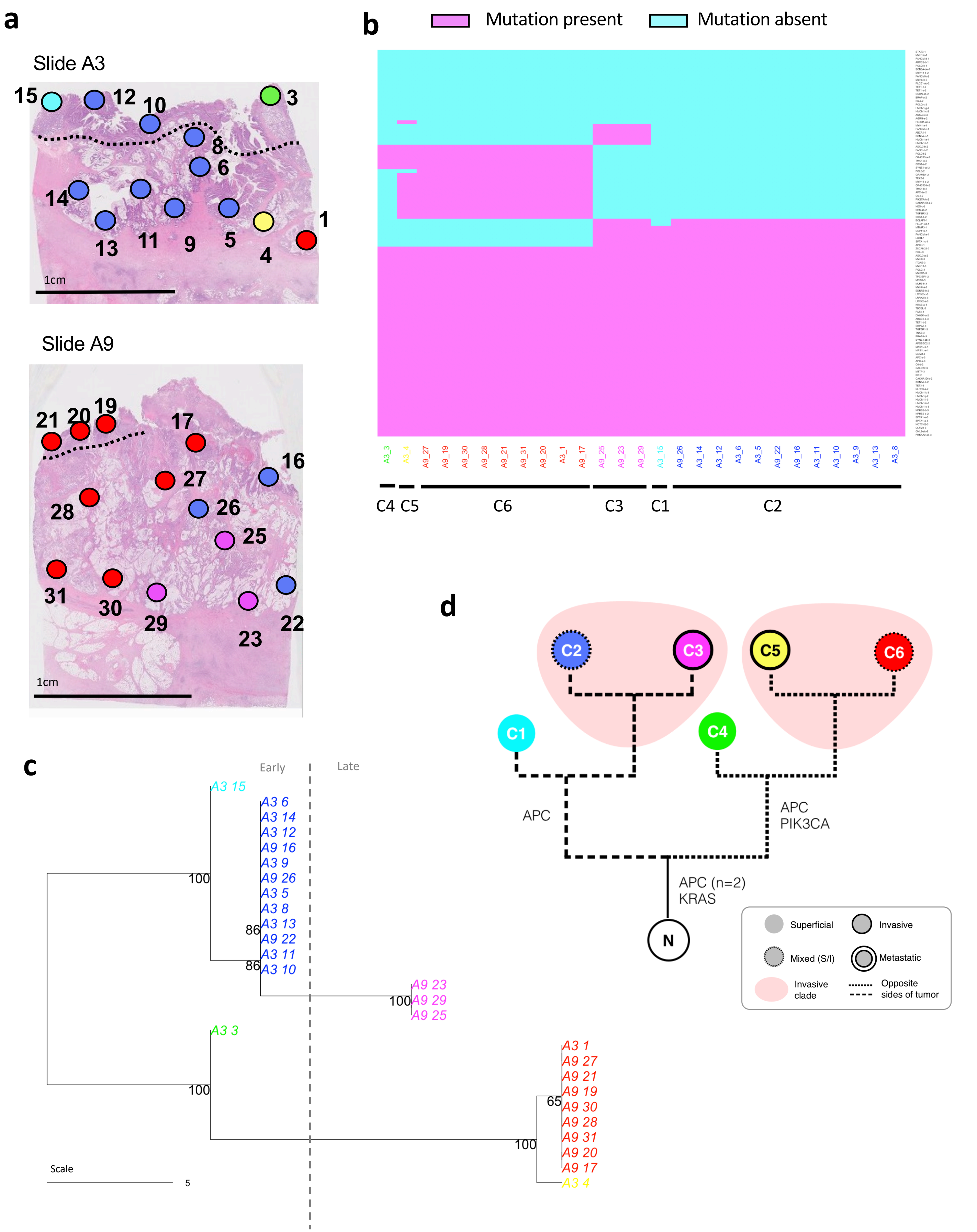

a
Slide A3
15
12
10
3
8
6
14
13
11
9
5
4
1
1cm
Slide A9
21
20
19
17
16
27
28
26
25
31
30
29
23
22
1cm
b
Mutation present
Mutation absent
A3_3
A3_4
A9_27
A9_19
A9_30
A9_28
A9_21
A9_31
A9_20
A3_1
A9_17
A9_25
A9_23
A9_29
A3_15
A9_26
A3_14
A3_12
A3_6
A3_5
A9_22
A9_16
A3_11
A3_10
A3_9
A3_13
A3_8
C4
C5
C6
C3
C1
C2
c
Early
Late
A3 15
A3 6
A3 14
A3 12
A9 16
A3 9
A9 26
A3 5
A3 8
A3 13
A9 22
A3 11
A3 10
A9 23
A9 29
A9 25
A3 3
A3 1
A9 27
A9 21
A9 19
A9 30
A9 28
A9 31
A9 20
A9 17
A3 4
100
86
86
100
100
65
100
Scale
5
d
C2
C3
C5
C6
C1
C4
APC
APC
PIK3CA
APC (n=2)
KRAS
N
Superficial
Invasive
Mixed (S/I)
Metastatic
Invasive clade
Opposite sides of tumor

Tumor F

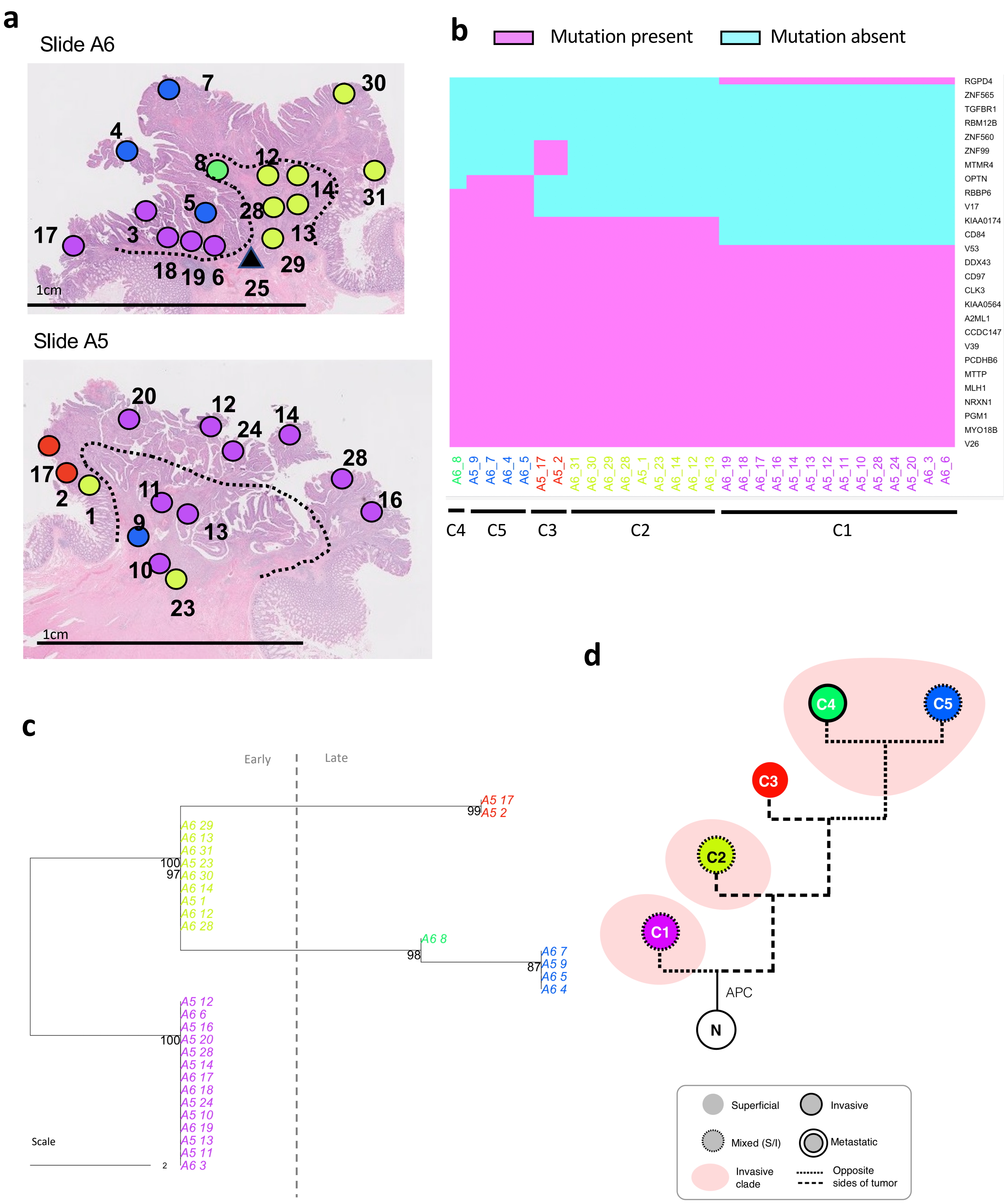

a
Slide A6
1cm
Slide A5
1cm
b
Mutation present
Mutation absent
RGPD4
ZNF565
TGFBR1
RBM12B
ZNF560
ZNF99
MTMR4
OPTN
RBBP6
V17
KIAA0174
CD84
V53
DDX43
CD97
CLK3
KIAA0564
A2ML1
CCDC147
V39
PCDHB6
MTTP
MLH1
NRXN1
PGM1
MYO18B
V26
C4
C5
C3
C2
C1
c
Early
Late
Scale
d
C4
C5
C3
C2
C1
APC
N
Superficial
Invasive
Mixed (S/I)
Metastatic
Invasive clade
Opposite sides of tumor

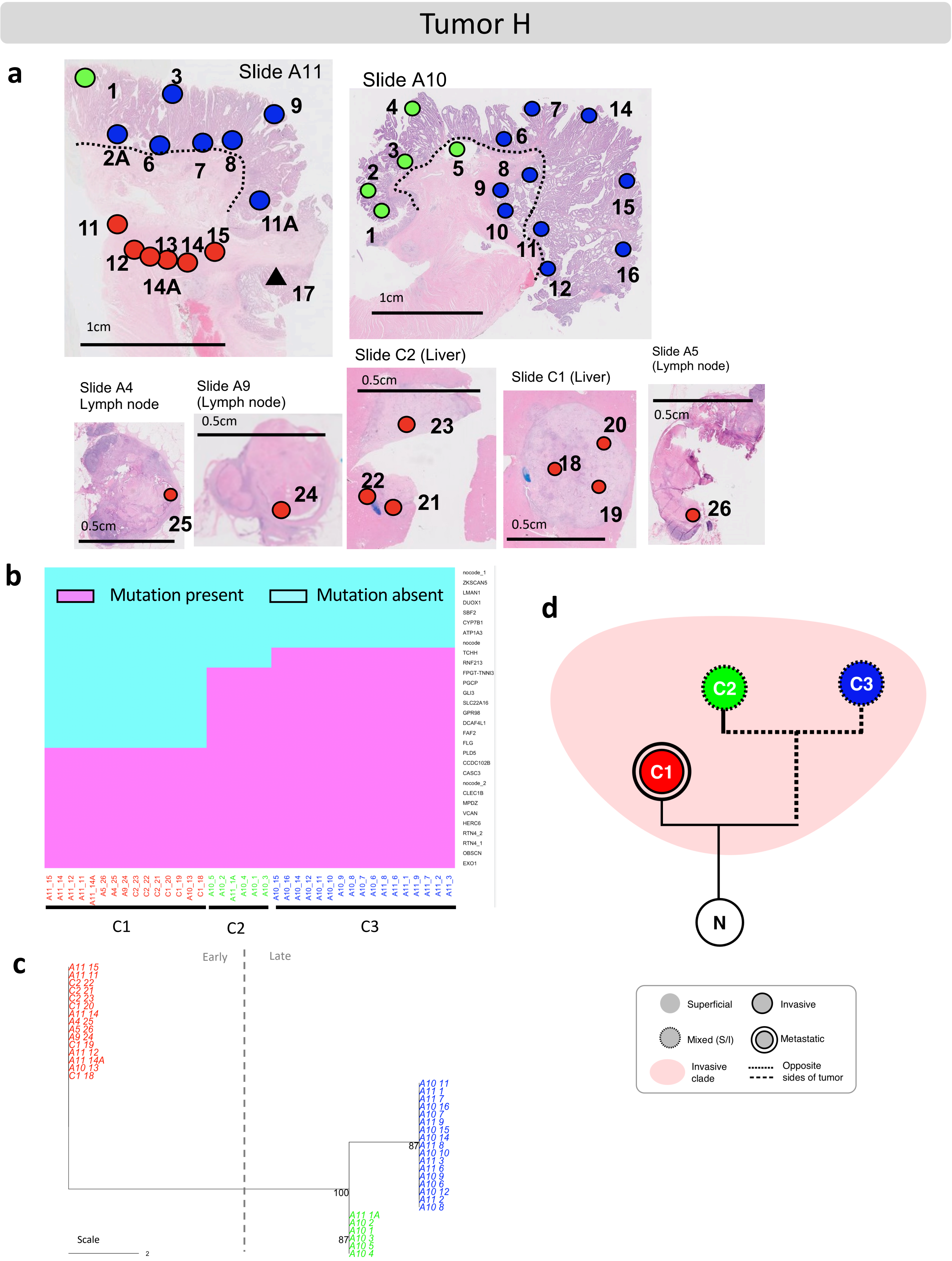

Tumor H
a
Slide A11
1cm
Slide A10
1cm
Slide C2 (Liver)
0.5cm
Slide C1 (Liver)
0.5cm
Slide A5
(Lymph node)
0.5cm
Slide A4
Lymph node
0.5cm
Slide A9
(Lymph node)
0.5cm
b
Mutation present
Mutation absent
nocode_1
ZKSCAN5
LMAN1
DUOX1
SBF2
CYP7B1
ATP1A3
nocode
TCHH
RNF213
FPGT-TNNI3
PGCP
GLI3
SLC22A16
GPR98
DCAF4L1
FAF2
FLG
PLD5
CCDC102B
CASC3
nocode_2
CLEC1B
MPDZ
VCAN
HERC6
RTN4_2
RTN4_1
OBSCN
EXO1
C1
C2
C3
c
Early
Late
Scale
2
d
C1
C2
C3
N
Superficial
Invasive
Mixed (S/I)
Metastatic
Invasive clade
Opposite sides of tumor

Tumor J

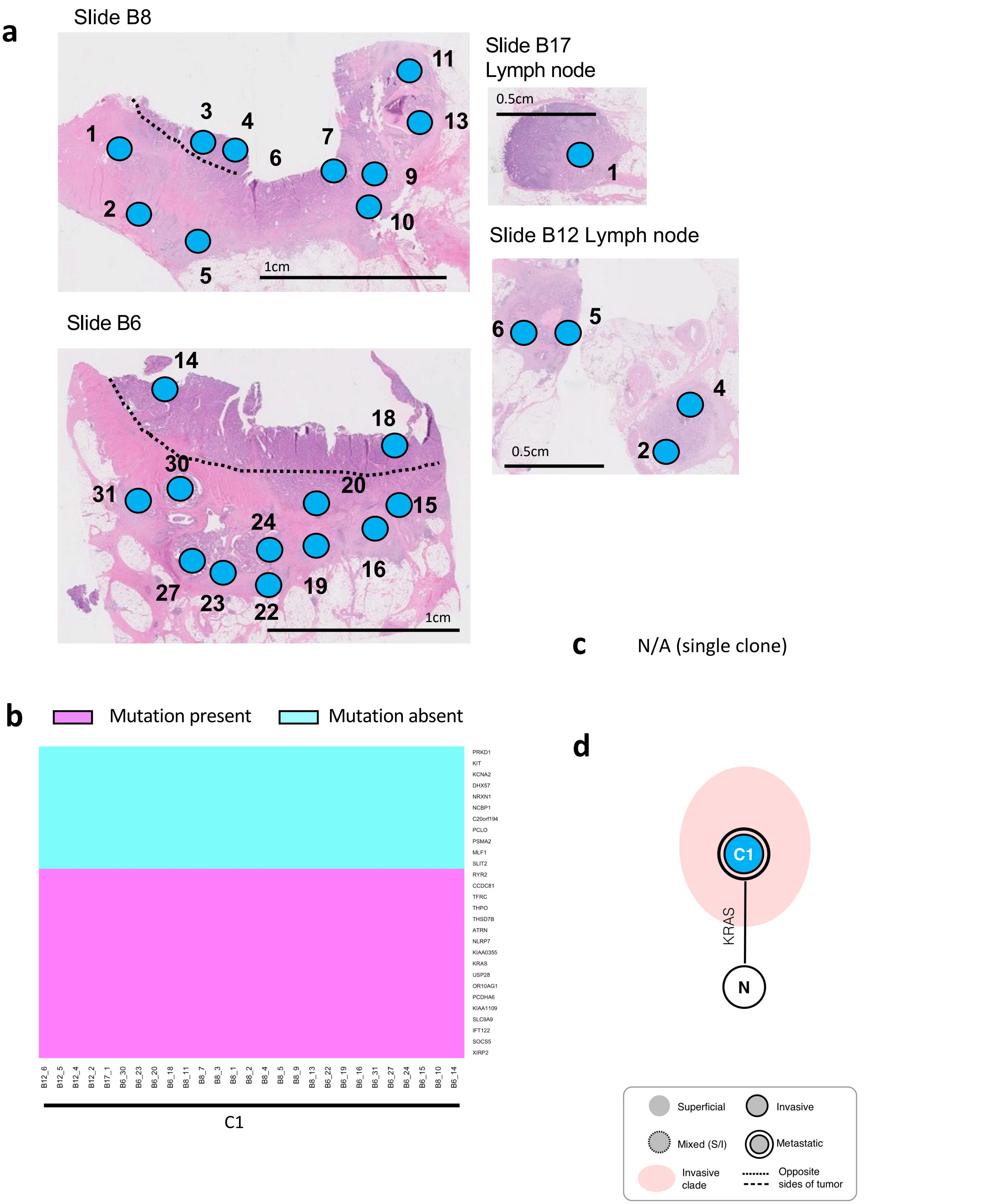

a
Slide B8
1cm
Slide B17
Lymph node
0.5cm
Slide B12 Lymph node
0.5cm
Slide B6
1cm
b
Mutation present
Mutation absent
PRKD1
KIT
KCNA2
DHX57
NRXN1
NCBP1
C20orf194
PCLO
PSMA2
MLF1
SLIT2
RYR2
CCDC81
TFRC
THPO
THSD7B
ATRN
NLRP7
KIAA0355
KRAS
USP28
OR10AG1
PCDHA6
KIAA1109
SLC9A9
IFT122
SOCS5
XIRP2
B12_6
B12_5
B12_4
B12_2
B17_1
B6_30
B6_23
B6_20
B6_18
B8_11
B8_7
B8_3
B8_1
B8_2
B8_4
B8_5
B8_9
B8_13
B6_22
B6_19
B6_16
B6_31
B6_27
B6_24
B6_15
B8_10
B6_14
C1
c
N/A (single clone)
d
C1
KRAS
N
Superficial
Invasive
Mixed (S/I)
Metastatic
Invasive clade
Opposite sides of tumor

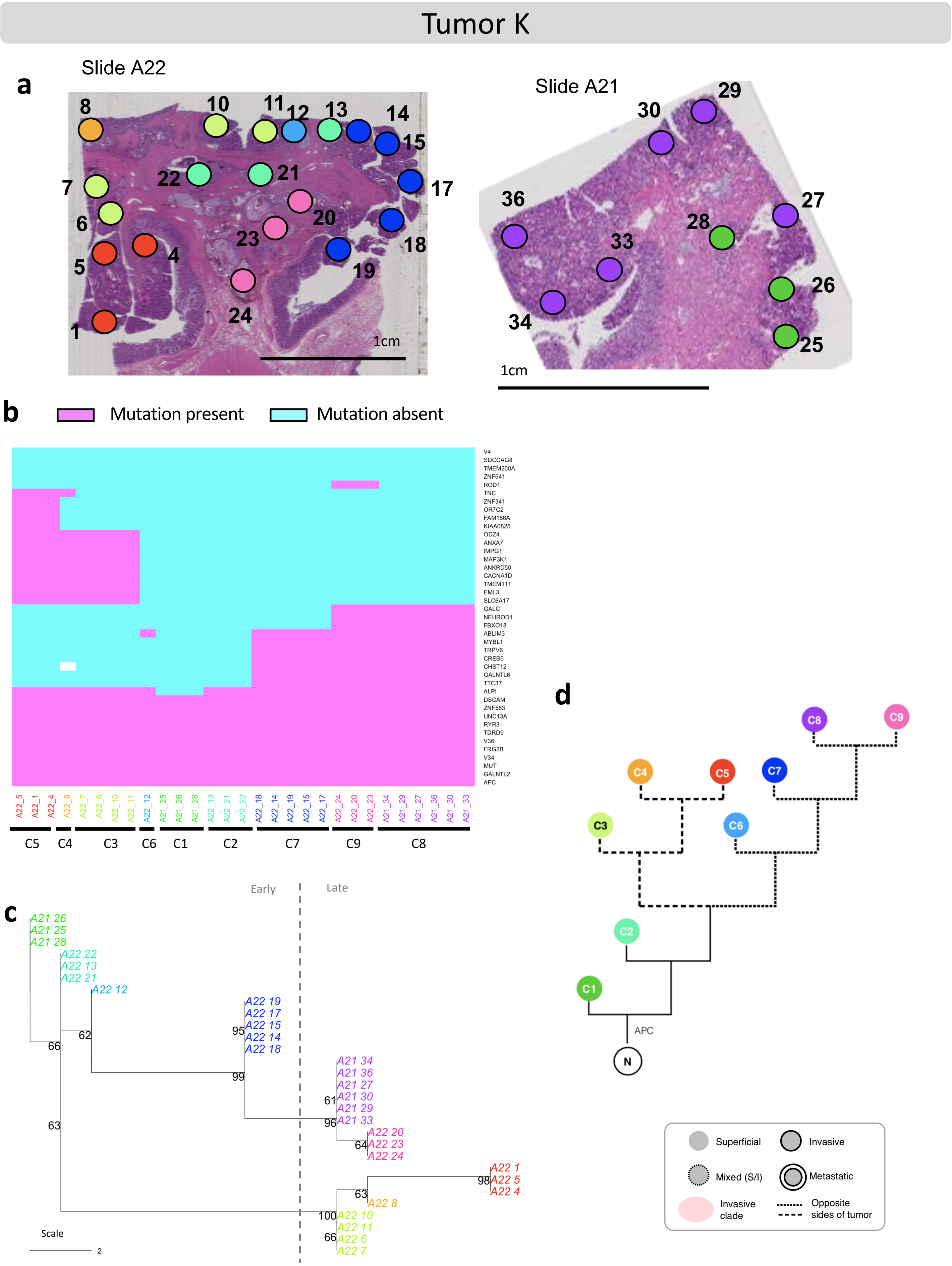

Tumor K
a
Slide A22
Slide A21
1cm
1cm
b
Mutation present
Mutation absent
V4
SDCCAG8
TMEM200A
ZNF641
ROD1
TNC
ZNF341
OR7C2
FAM186A
KIAA0825
ODZ4
ANXA7
IMPG1
MAP3K1
ANKRD50
CACNA1D
TMEM111
EML3
SLC6A17
GALC
NEUROD1
FBXO18
ABLIM3
MYBL1
TRPV6
CREB5
CHST12
GALNTL6
TTC37
ALPI
DSCAM
ZNF583
UNC13A
RYR3
TDRD9
V36
FRG2B
V34
MUT
GALNTL2
APC
A22_5 A22_1 A22_4 A22_8 A22_7 A22_6 A22_10 A22_11 A22_12 A21_25 A21_26 A21_28 A22_13 A22_21 A22_22 A22_18 A22_14 A22_19 A22_15 A22_17 A22_24 A22_20 A22_23 A21_34 A21_29 A21_27 A21_36 A21_30 A21_33
C5 C4 C3 C6 C1 C2 C7 C9 C8
c
Early
Late
Scale
2
d
C1 C2 C3 C4 C5 C6 C7 C8 C9
APC
N
Superficial
Invasive
Mixed (S/I)
Metastatic
Invasive clade
Opposite sides of tumor

Tumor M

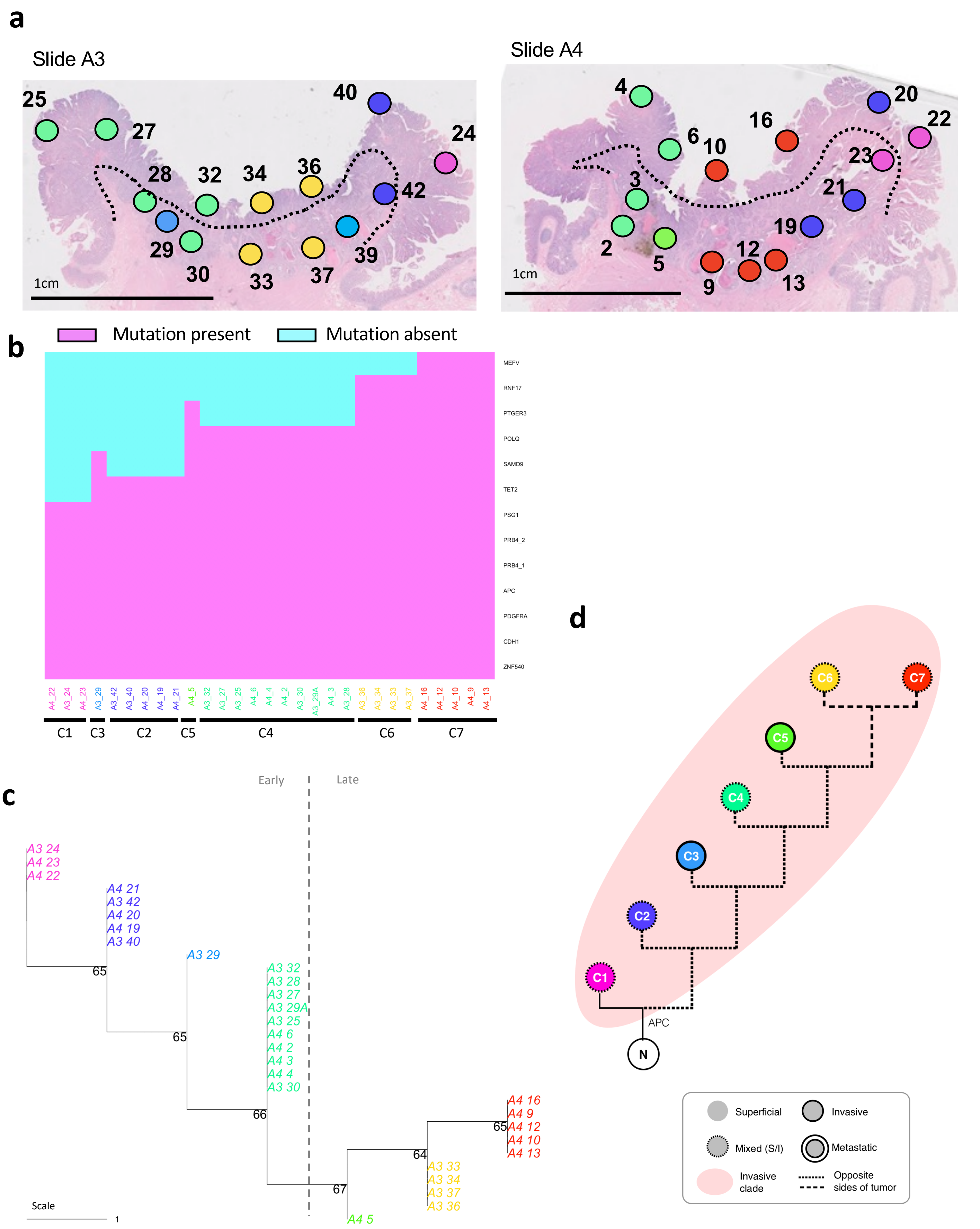
a
Slide A3
25
27
40
24
28
32
34
36
42
29
30
33
37
39
1cm
Slide A4
4
6
10
16
20
22
23
3
21
19
2
5
9
12
13
1cm
b
Mutation present
Mutation absent
MEFV
RNF17
PTGER3
POLQ
SAMD9
TET2
PSG1
PRB4_2
PRB4_1
APC
PDGFRA
CDH1
ZNF540
A4_22
A3_24
A4_23
A3_29
A3_42
A3_40
A4_20
A4_19
A4_21
A4_5
A3_32
A3_27
A3_25
A4_6
A4_4
A4_2
A3_30
A3_29A
A4_3
A3_28
A3_36
A3_34
A3_33
A3_37
A4_16
A4_12
A4_10
A4_9
A4_13
C1
C3
C2
C5
C4
C6
C7
c
Early
Late
A3 24
A4 23
A4 22
A4 21
A3 42
A4 20
A4 19
A3 40
A3 29
A3 32
A3 28
A3 27
A3 29A
A3 25
A4 6
A4 2
A4 3
A4 4
A3 30
A4 16
A4 9
A4 12
A4 10
A4 13
A3 33
A3 34
A3 37
A3 36
A4 5
65
65
66
67
64
65
Scale
1
d
C6
C7
C5
C4
C3
C2
C1
APC
N
Superficial
Invasive
Mixed (S/I)
Metastatic
Invasive clade
Opposite sides of tumor

Tumor R

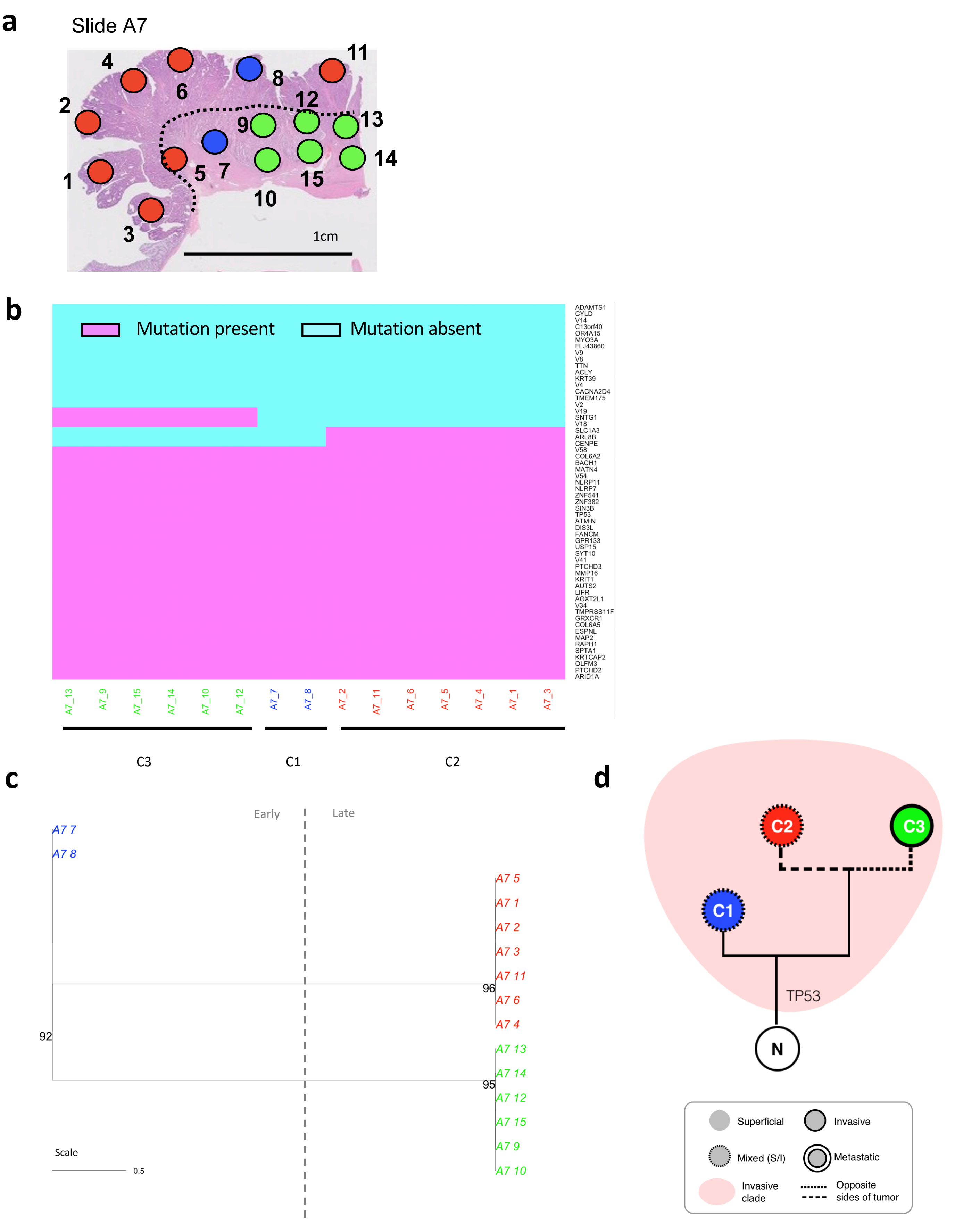
a
Slide A7
1cm
b
Mutation present
Mutation absent
ADAMTS1
CYLD
V14
C13orf40
OR4A15
MYO3A
FLJ43860
V9
V8
TTN
ACLY
KRT39
V4
CACNA2D4
TMEM175
V2
V19
SNTG1
V18
SLC1A3
ARL8B
CENPE
V58
COL6A2
BACH1
MATN4
V54
NLRP11
NLRP7
ZNF541
ZNF382
SIN3B
TP53
ATMIN
DIS3L
FANCM
GPR133
USP15
SYT10
V41
PTCHD3
MMP16
KRIT1
AUTS2
LIFR
AGXT2L1
V34
TMPRSS11F
GRXCR1
COL6A5
ESPNL
MAP2
RAPH1
SPTA1
KRTCAP2
OLFM3
PTCHD2
ARID1A
A7_13
A7_9
A7_15
A7_14
A7_10
A7_12
A7_7
A7_8
A7_2
A7_11
A7_6
A7_5
A7_4
A7_1
A7_3
C3
C1
C2
c
Early
Late
A7 7
A7 8
A7 5
A7 1
A7 2
A7 3
A7 11
A7 6
A7 4
A7 13
A7 14
A7 12
A7 15
A7 9
A7 10
96
92
95
Scale
0.5
d
C2
C3
C1
TP53
N
Superficial
Invasive
Mixed (S/I)
Metastatic
Invasive clade
Opposite sides of tumor

Tumor T

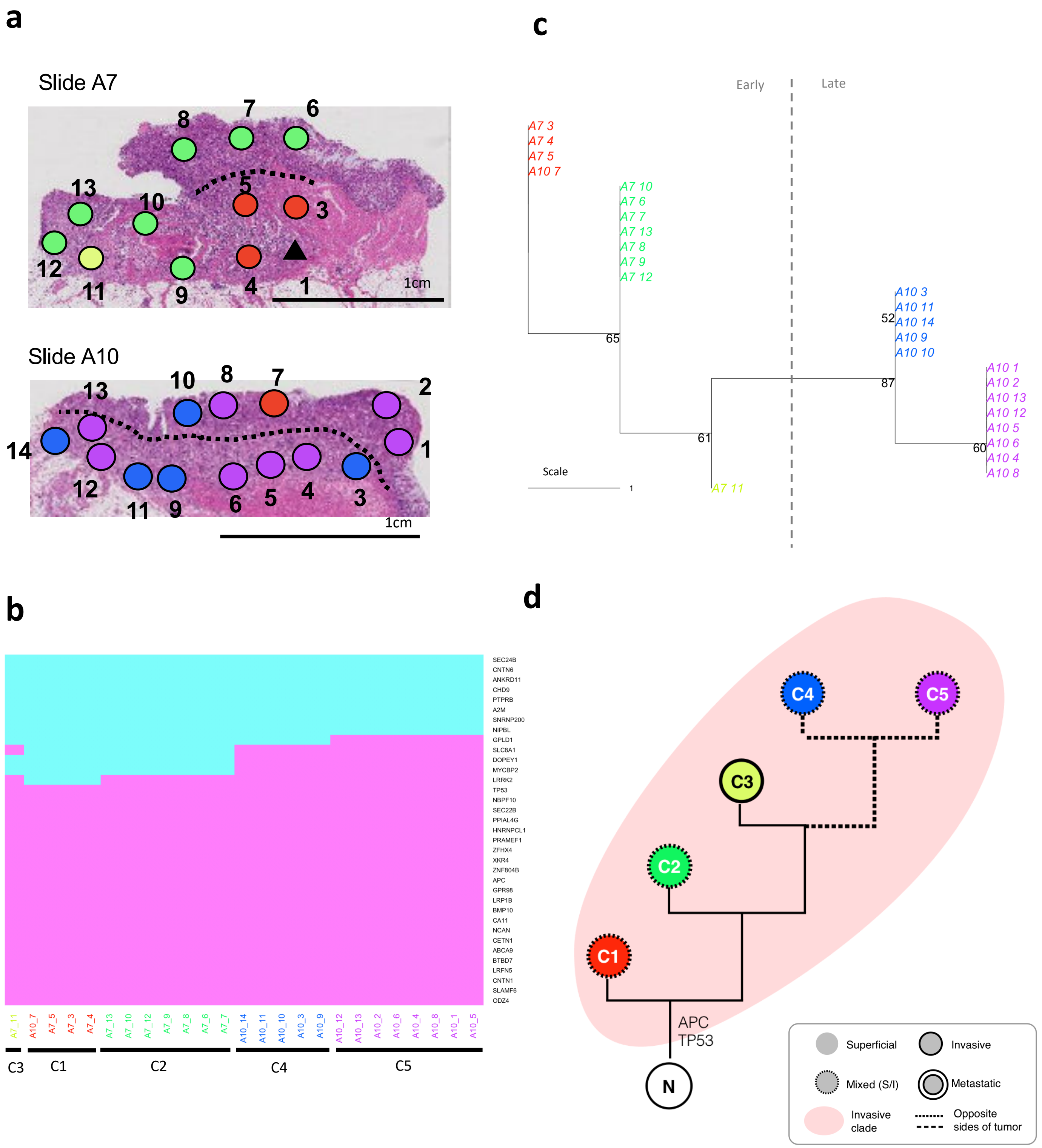

a
Slide A7
1cm
Slide A10
1cm
b
SEC24B
CNTN6
ANKRD11
CHD9
PTPRB
A2M
SNRNP200
NIPBL
GPLD1
SLC8A1
DOPEY1
MYCBP2
LRRK2
TP53
NBPF10
SEC22B
PPIAL4G
HNRNPCL1
PRAMEF1
ZFHX4
XKR4
ZNF804B
APC
GPR98
LRP1B
BMP10
CA11
NCAN
CETN1
ABCA9
BTBD7
LRFN5
CNTN1
SLAMF6
ODZ4
C3
C1
C2
C4
C5
c
Early
Late
Scale
d
APC
TP53
N
C1
C2
C3
C4
C5
Superficial
Invasive
Mixed (S/I)
Metastatic
Invasive clade
Opposite sides of tumor

Tumor U

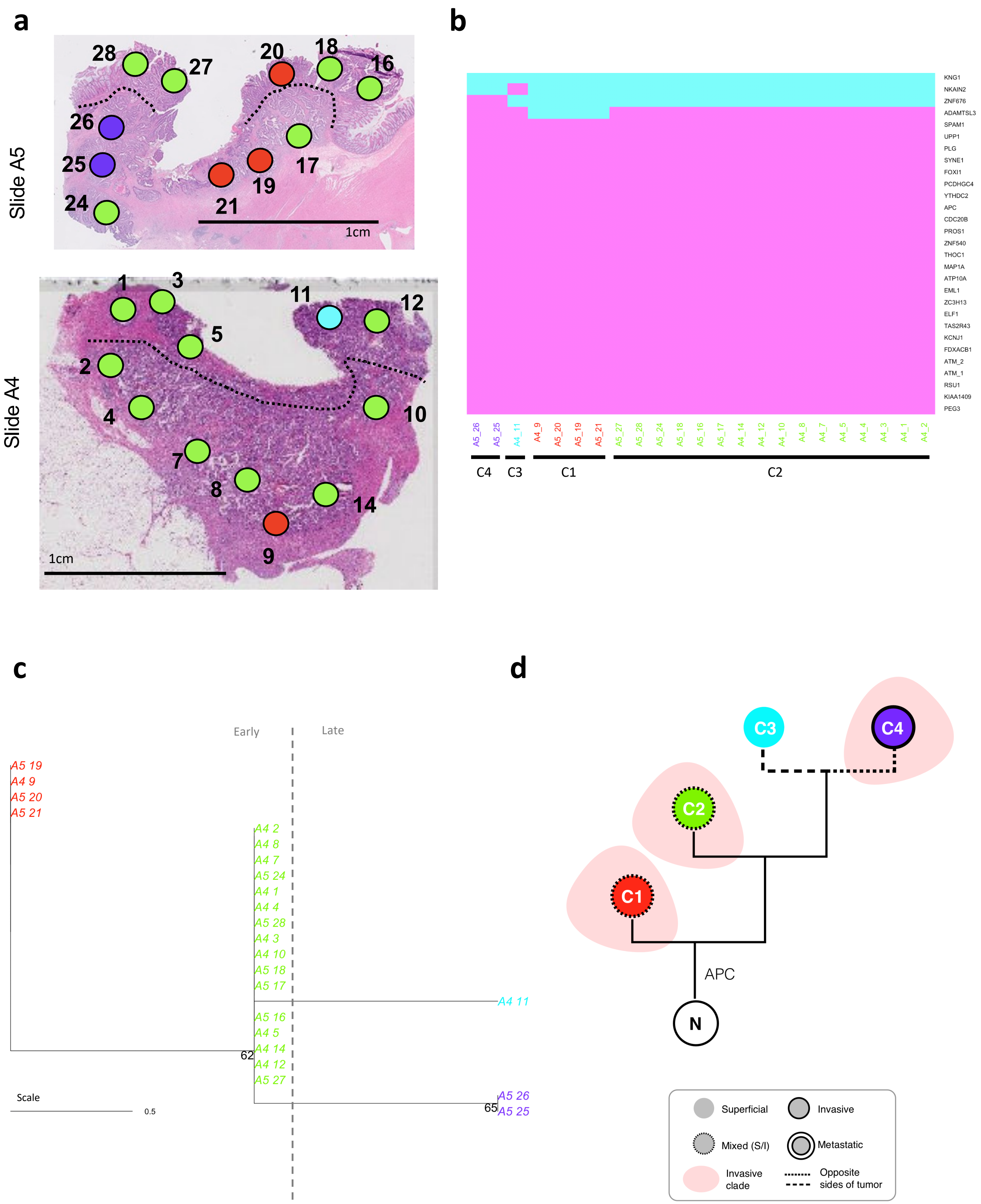
a
Slide A5
Slide A4
1cm
1cm
b
KNG1
NKAIN2
ZNF676
ADAMTSL3
SPAM1
UPP1
PLG
SYNE1
FOXI1
PCDHGC4
YTHDC2
APC
CDC20B
PROS1
ZNF540
THOC1
MAP1A
ATP10A
EML1
ZC3H13
ELF1
TAS2R43
KCNJ1
FDXACB1
ATM_2
ATM_1
RSU1
KIAA1409
PEG3
C4
C3
C1
C2
c
Early
Late
A5 19
A4 9
A5 20
A5 21
A4 2
A4 8
A4 7
A5 24
A4 1
A4 4
A5 28
A4 3
A4 10
A5 18
A5 17
A4 11
A5 16
A4 5
A4 14
A4 12
A5 27
A5 26
A5 25
62
65
Scale
0.5
d
C3
C4
C2
C1
APC
N
Superficial
Invasive
Mixed (S/I)
Metastatic
Invasive clade
Opposite sides of tumor

Tumor W

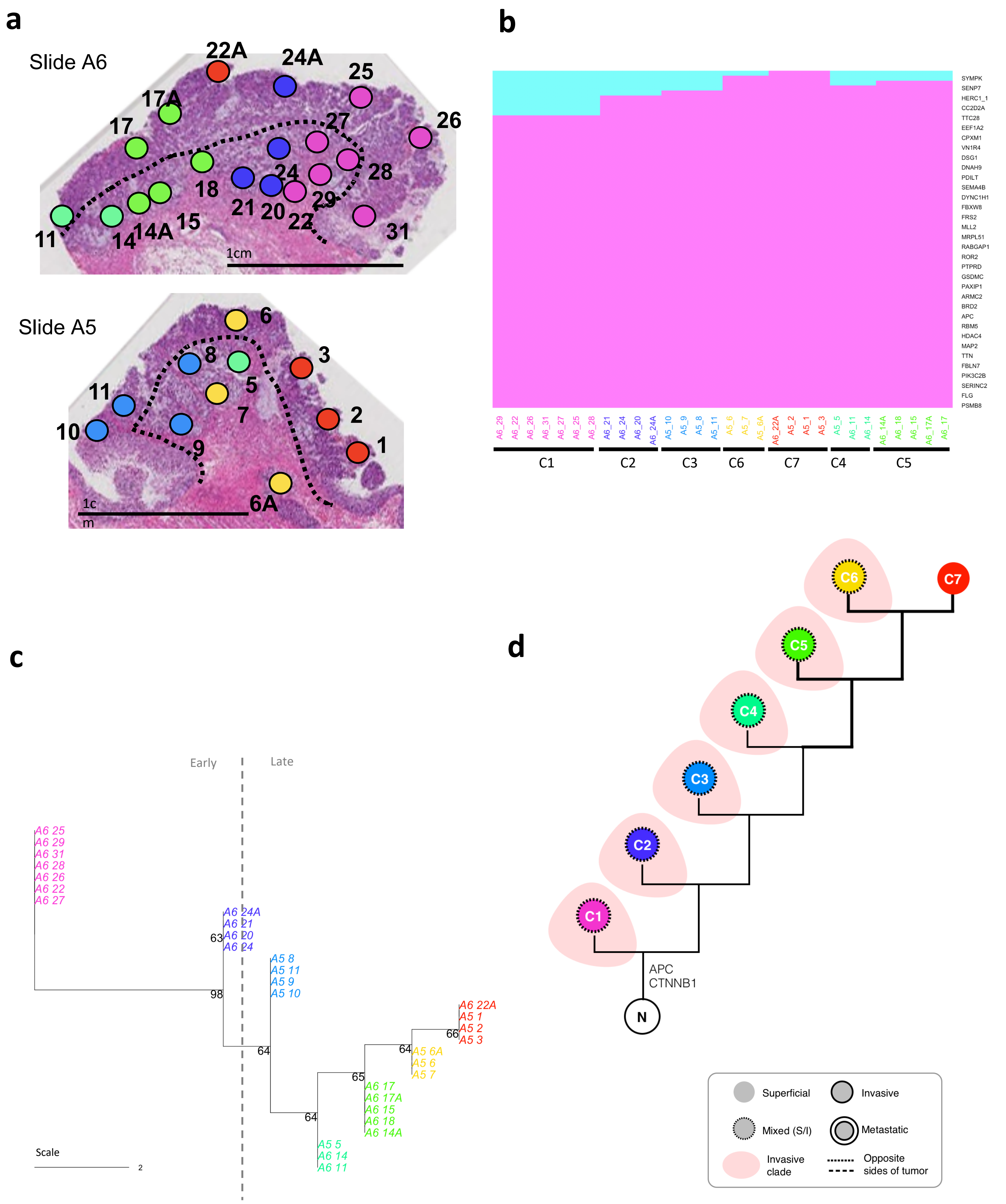
a
Slide A6
22A
24A
25
17A
17
27
26
28
24
18
21
20
29
22
11
14
14A
15
31
1cm
Slide A5
6
8
3
11
5
7
2
10
9
1
6A
1cm
b
SYMPK
SENP7
HERC1_1
CC2D2A
TTC28
EEF1A2
CPXM1
VN1R4
DSG1
DNAH9
PDILT
SEMA4B
DYNC1H1
FBXW8
FRS2
MLL2
MRPL51
RABGAP1
ROR2
PTPRD
GSDMC
PAXIP1
ARMC2
BRD2
APC
RBM5
HDAC4
MAP2
TTN
FBLN7
PIK3C2B
SERINC2
FLG
PSMB8
A6_29 A6_22 A6_26 A6_31 A6_27 A6_25 A6_28
A6_21 A6_24 A6_20 A6_24A
A5_10 A5_9 A5_8 A5_11
A5_6 A5_7 A5_6A
A6_22A A5_2 A5_1 A5_3
A5_5 A6_11 A6_14
A6_14A A6_18 A6_15 A6_17A A6_17
C1
C2
C3
C6
C7
C4
C5
c
Early
Late
A6 25
A6 29
A6 31
A6 28
A6 26
A6 22
A6 27
A6 24A
A6 21
A6 20
A6 24
63
98
A5 8
A5 11
A5 9
A5 10
64
A6 22A
A5 1
A5 2
A5 3
66
64
A5 6A
A5 6
A5 7
65
A6 17
A6 17A
A6 15
A6 18
A6 14A
64
A5 5
A6 14
A6 11
Scale
2
d
C6
C7
C5
C4
C3
C2
C1
APC
CTNNB1
N
Superficial
Invasive
Mixed (S/I)
Metastatic
Invasive clade
Opposite sides of tumor

Supplementary Figure 5. Example of the SURF protocol. DNA was isolated from tumor cells underneath the black ink dots on the lightly stained tissue section mounted on a plastic slide after shortwave ultraviolet irradiation. The stained H&E section of Tumor H is shown for comparison.

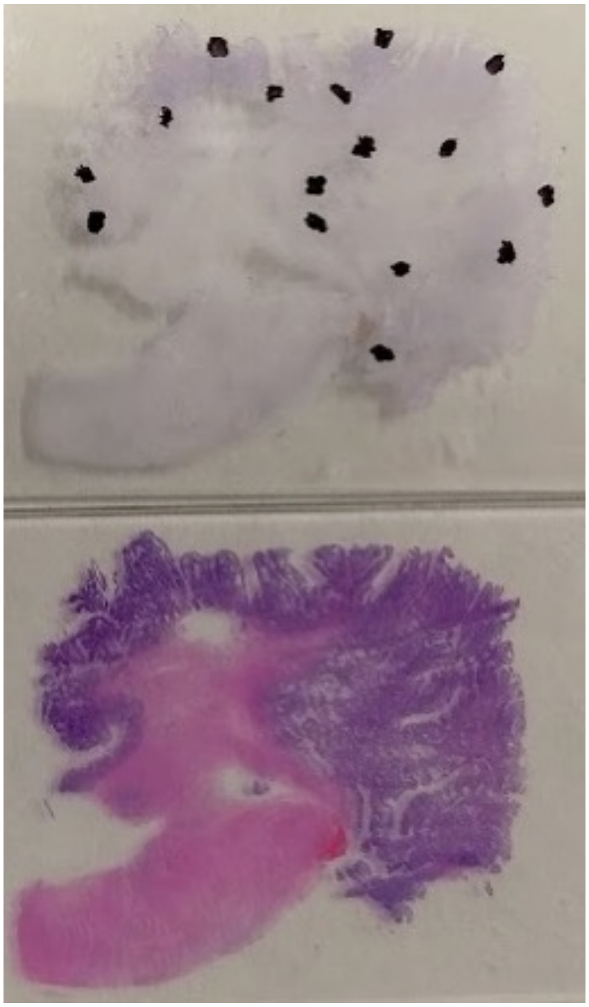

Supplementary Figure 6. Correlation between physical and genetic distance. Tumor-specific Pearson correlation between genetic and physical distances ranged from -0.2 to 0.8 (median: 0.37).

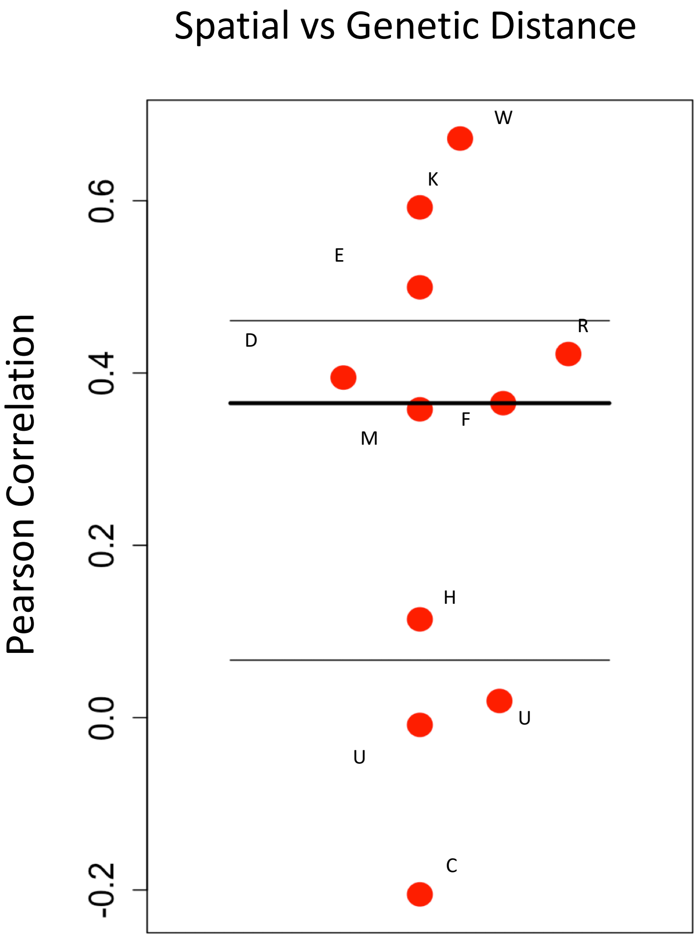

Supplementary Figure 7. Within- and between-slide subclone comparison. For each tumor, pairwise genetic distances were calculated between subclones in the same slide (within slide) and between subclones of separate slides (between slides). The two groups were compared using a two-sided Wilcoxon rank sum test and p-values are shown in the subplot titles.

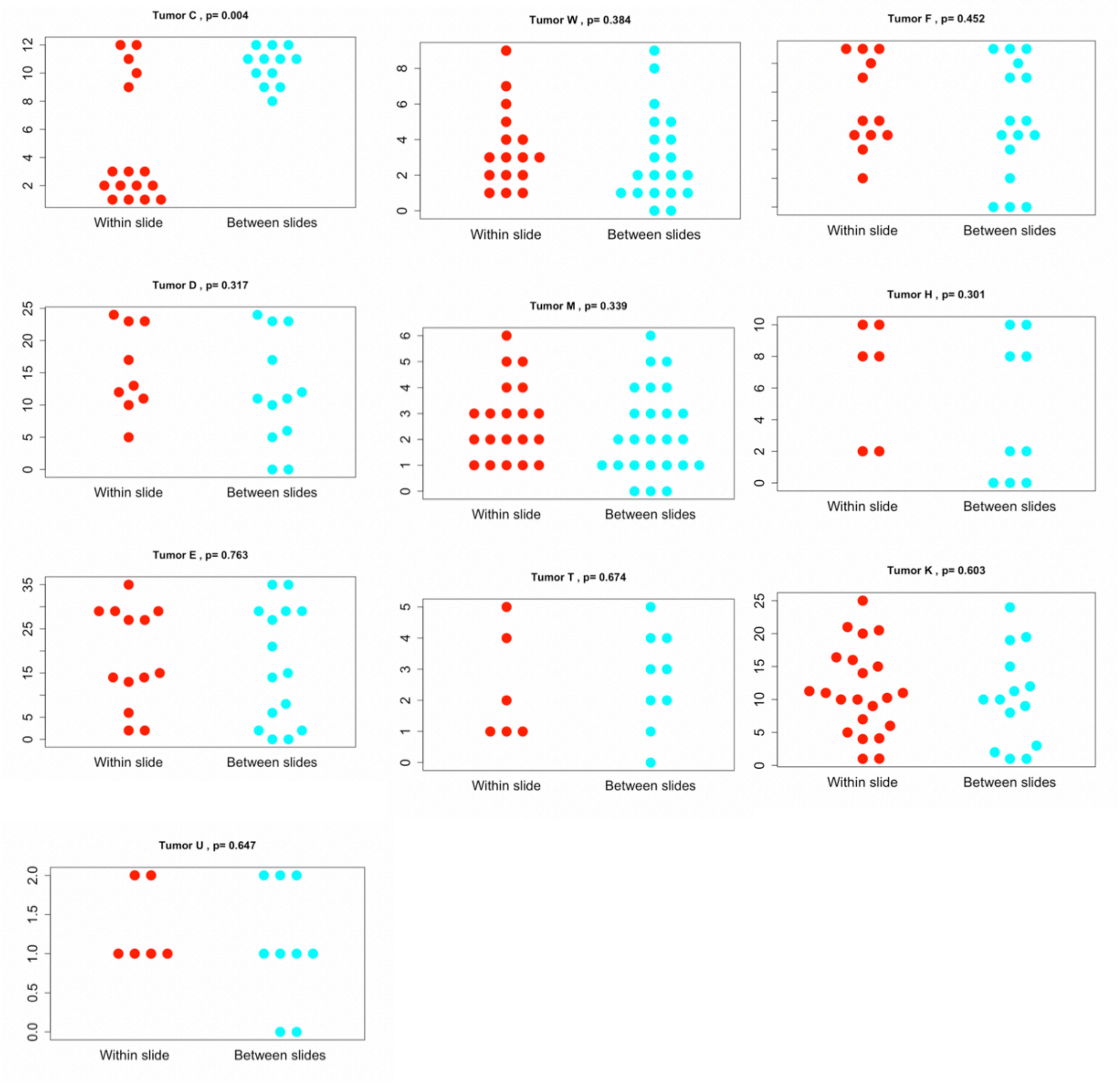

Supplementary References

1 Ryser, M. D., Min, B. H., Siegmund, K. D. & Shibata, D. Spatial mutation patterns as markers of early colorectal tumor cell mobility. *Proc Natl Acad Sci U S A* **115**, 5774-5779, doi:10.1073/pnas.1716552115 (2018).
