# Supplementary material for: Minimal barriers to invasion during human colorectal tumor growth
Source: Nat Commun. 2020 Mar 9;11:1280. doi: 10.1038/s41467-020-14908-7 (PMC7062901; doi:10.1038/s41467-020-14908-7)
Supplement: Supplementary file 3 — Description of Additional Supplementary Files [file 41467_2020_14908_MOESM3_ESM.pdf]

Description of Additional Supplementary Files

File Name: Supplementary Data 1

Description: For each tumor (X), there are three sheets. The first sheet (tumor_X_gen), contains genetic information in the form of the minor allele frequency (MAF) for each sequenced single nucleotide variant, in all microdissected spots (columns); phenotype abbreviations as follows: inv=invasive, super=superficial, met=metastasis. The second (tumor_X_distance_slide1) and third (tumor_X_distance_slide2) sheets contain the pairwise physical distances between the spots within slide1 and slide 2, respectively.
